# Conbercept Treatment for Heart‐Shaped Vascular Intertwined Nets in Macular Neovascularization: Anti‐VEGF Drug Therapy Strategy Based on Vascular Geometry Diagnosed by OCTA

**DOI:** 10.1002/ccr3.71250

**Published:** 2025-10-21

**Authors:** Shangkun Ou, Feiping Shi, Minqing Cai, Yiming Wu

**Affiliations:** ^1^ Department of Ophthalmology The Affiliated Hospital of Guizhou Medical University Guiyang Guizhou China; ^2^ Xiamen Eye Center and Eye Institute of Xiamen University, School of Medicine Xiamen China; ^3^ Fujian Provincial Key Laboratory of Ophthalmology and Visual Science, Fujian Engineering and Research Center of Eye Regenerative Medicine Xiamen University Xiamen Fujian China; ^4^ China Scholarship Council Beijing China; ^5^ Department of Biomedical Sciences, School of Infection, Inflammation and Immunology, College of Medicine and Health University of Birmingham Birmingham United Kingdom

**Keywords:** artificial intelligence, choroidal neovascularization, Conbercept, macular neovascularization, optical coherence tomography angiography

## Abstract

Macular neovascularization (MNV), a hallmark of several retinal disorders including ocular trauma and wet age‐related macular degeneration, remains a major cause of vision impairment due to the proliferation of abnormal, fragile blood vessels. Anti‐VEGF therapies, such as Aflibercept (Eylea), Bevacizumab (Avastin), Brolucizumab (Beovu), Conbercept (Lumitin), Faricimab (Vabysmo), Ranibizumab (Lucentis), and Pegaptanib (Macugen), have significantly transformed MNV management, targeting VEGF to curb this pathological vascular growth. In this report, we describe a 63‐year‐old male with a history of hypertension and diabetes who developed acute vision loss attributed to MNV secondary to hypertensive retinopathy. Optical coherence tomography angiography (OCTA) revealed unusual heart‐shaped, intertwined vascular nets, informing the choice of Conbercept for intravitreal injection. This personalized therapeutic decision led to marked visual improvement over a 12‐month period. The case exemplifies the importance of vascular geometry in guiding anti‐VEGF selection, supported by existing literature that links specific neovascular geometries to differential drug responsiveness. Conbercept, in particular, proved effective against the complex intertwined nets observed in this patient. These findings emphasize the promise of individualized treatment strategies and the potential of OCTA‐based vascular geometry classification as a tool for precision medicine.

## Graphical abstract

A 63‐year‐old male with a history of hypertension and diabetes developed acute vision loss, which was attributed to macular neovascularization secondary to hypertensive retinopathy. Optical coherence tomography angiography revealed unusual heart‐shaped, intertwined vascular nets, informing the choice of Conbercept for intravitreal injection. This personalized therapeutic decision led to marked visual improvement over a 12‐month period.

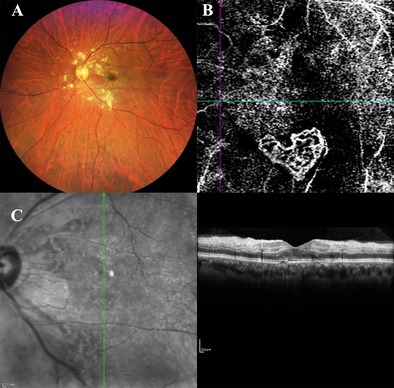

**Summary**

- OCTA‐based vascular geometry classification enables personalized anti‐VEGF treatment in MNV.
- Conbercept is effective for intertwined net‐type MNV, improving vision over 1 year.

**Abbreviations**
AI: artificial intelligence
anti‐VEGF: anti‐vascular endothelial growth factor
BCVA: best‐corrected visual acuity
CNV: choroidal neovascularization
DME: diabetic macular edema
FDA: the U.S. Food and Drug Administration
MNV: macular neovascularization
NMPA: National Medical Products Administration
OCTA: optical coherence tomography angiography
wAMD: wet age‐related macular degeneration

## 1 Introduction

Macular neovascularization (MNV) is a pathological feature present in various chorioretinal conditions, such as pathological myopia and ocular trauma, though it is most frequently associated with wet age‐related macular degeneration (wAMD) [1]. In the majority of cases, MNV originates from the choroidal vasculature (choroidal neovascularization, CNV), extending either beneath the retinal pigment epithelium (type 1 MNV) or into the subretinal space (type 2 MNV). More recently, it has been established that type 3 neovascular age‐related macular degeneration, also known as retinal angiomatous proliferation, emerges from the deep retinal capillary plexus and advances outward to form vascular connections with the choriocapillaris, characterizing type 3 MNV [2]. These newly formed vessels are structurally fragile and inherently prone to leakage, leading to the extravasation of blood, lipids, and fluid beneath the retina [3]. This pathological accumulation of fluid and blood beneath the retina often results in swelling, particularly in the macular region, a condition referred to as macular edema. The presence of macular edema exerts significant mechanical stress on the delicate retinal tissue and may lead to further complications such as hemorrhaging and the formation of fibrotic scars. These subsequent pathological changes severely disrupt the normal architecture and function of retinal cells, which are critical for visual acuity, ultimately leading to progressive vision loss.

In response to the significant burden imposed by these retinal diseases, extensive research and development efforts have been directed toward therapies that can effectively slow disease progression and preserve vision [4, 5, 6, 7, 8, 9, 10, 11]. A major breakthrough in the management of ocular vascular diseases has been the development and clinical introduction of anti‐vascular endothelial growth factor (anti‐VEGF) therapies. VEGF is not only one of the most critical molecules in the progression of MNV but also plays a key role in the growth of neovascularization in most ocular vascular diseases, such as corneal neovascularization, iris neovascularization, and anterior uveitis, often accompanied by other inflammatory mediators and pro‐angiogenic molecules [12, 13, 14, 15, 16]. To date, approximately seven anti‐VEGF drugs have received approval from regulatory bodies such as the U.S. Food and Drug Administration (FDA) or the National Medical Products Administration (NMPA) and are now utilized in ophthalmology clinics across various countries (Table 1). These drugs include Aflibercept (Eylea), Bevacizumab (Avastin), Brolucizumab (Beovu), Conbercept (Lumitin), Faricimab (Vabysmo), Ranibizumab (Lucentis), and Pegaptanib (Macugen) [17, 18, 19]. It is worth noting that Pegaptanib, although initially a pioneering anti‐VEGF therapy, has almost completely exited the market due to the emergence of newer, more effective agents [20]. With the exception of Bevacizumab, which was originally developed and approved for the treatment of metastatic colorectal cancer, the remaining six drugs were specifically designed to target and inhibit the process of neovascularization in wAMD and other related ocular diseases. These anti‐VEGF drugs have revolutionized the treatment landscape, offering patients the possibility of maintaining their vision and improving their quality of life.

**TABLE 1:** The current approved anti‐VEGF drugs for ocular diseases in clinic.

Drug name^a^	Brand name	First approval	Approval agency	Primary indication	Drug information	Potential target of vascular geometry
Bevacizumab	Avastin	2004	FDA	Metastatic colorectal cancer	Fully humanized monoclonal antibody, inhibits all forms of VEGF. Off‐label for ocular use in most regions.	MNV forms distinct clusters that exhibit discontinuity from the surrounding perifoveal vessels in OCTA imaging
Pegaptanib	Macugen	2004	FDA	wAMD	RNA aptamer, selectively binds and blocks VEGF165	/
Ranibizumab	Lucentis	2006	FDA	wAMD	Anti‐VEGF antibody fragment, specifically designed for ocular use	Tree‐like MNV
Aflibercept	Eylea	2011	FDA	wAMD	Fusion protein, binds and blocks VEGF‐A, VEGF‐B, and PlGF	Glomerular‐like MNV
Conbercept	Lumitin	2013	NMPA	wAMD	Fusion protein, binds and blocks VEGF‐A, VEGF‐B, and PlGF	Intertwined nets; A large, highly organized high‐flow vascular network with intricate capillary branches, encircled by a dark halo and accompanied by a feeder vessel
Brolucizumab	Beovu	2019	FDA	wAMD	Humanized single‐chain antibody fragment	/
Faricimab	Vabysmo	2022	FDA	wAMD, DME	Bispecific antibody, inhibits both VEGF‐A and Ang‐2	/

^a^ Their biosimilars are not included.

Given the diverse causes and presentations of MNV, the development and implementation of anti‐VEGF therapies have been tailored to target the specific mechanisms driving neovascularization. Consequently, a number of comparative clinical trials have been conducted to determine the most effective anti‐VEGF agents for treating different types of MNV, based on both the classification of the condition and the treatment mechanisms of the drugs. For instance, in patients with myopic MNV, clinical studies have demonstrated that Ranibizumab is particularly effective, as it rapidly reduces MNV‐induced central macular thickening with fewer injections required compared to Bevacizumab [21]. In cases of polypoidal MNV [22] or wAMD [23, 24], Brolucizumab has shown superior efficacy in resolving retinal fluid compared to Aflibercept, highlighting its potential as a preferred option in these conditions. Additionally, in the management of diabetic macular edema (DME), particularly among patients with poor baseline best‐corrected visual acuity (BCVA ≤ 69 letters), Ranibizumab has been found to more effectively reduce central retinal thickness and improve visual acuity compared to Bevacizumab [25]. Another similar study indicated that Aflibercept outperformed Ranibizumab and Bevacizumab [26]. The findings from these comparative studies are instrumental in guiding ophthalmologists when selecting the most appropriate anti‐VEGF therapy for individual patients, taking into account the specific characteristics and underlying causes of their MNV. For example, new approaches have begun exploring how the morphological geometry of MNV might inform the selection of anti‐VEGF agents [27, 28, 29, 30, 31]. This is problematic because, clinically, there is a pressing need to enhance the decision‐making process for anti‐VEGF treatment initiation and the re‐treatment of MNV based on the response [31, 32]. Analogous systematic reviews from other vascular systems, such as the carotid artery, show that vessel geometry is closely related to hemodynamic patterns, which in turn influence disease progression and treatment outcomes [33]. These parallels highlight the potential value of integrating morphological geometry into the therapeutic decision‐making process for MNV. Furthermore, at our clinic, we have observed a particularly instructive case of MNV in which the optimal anti‐VEGF treatment recommendation was successfully determined based on distinct vascular geometry identified through optical coherence tomography angiography (OCTA). This case report underscores the importance of personalized medicine in ophthalmology, where detailed imaging and classification of MNV can lead to more targeted and effective therapeutic interventions.

## 2 Case History/Examination

**FIGURE 1:**
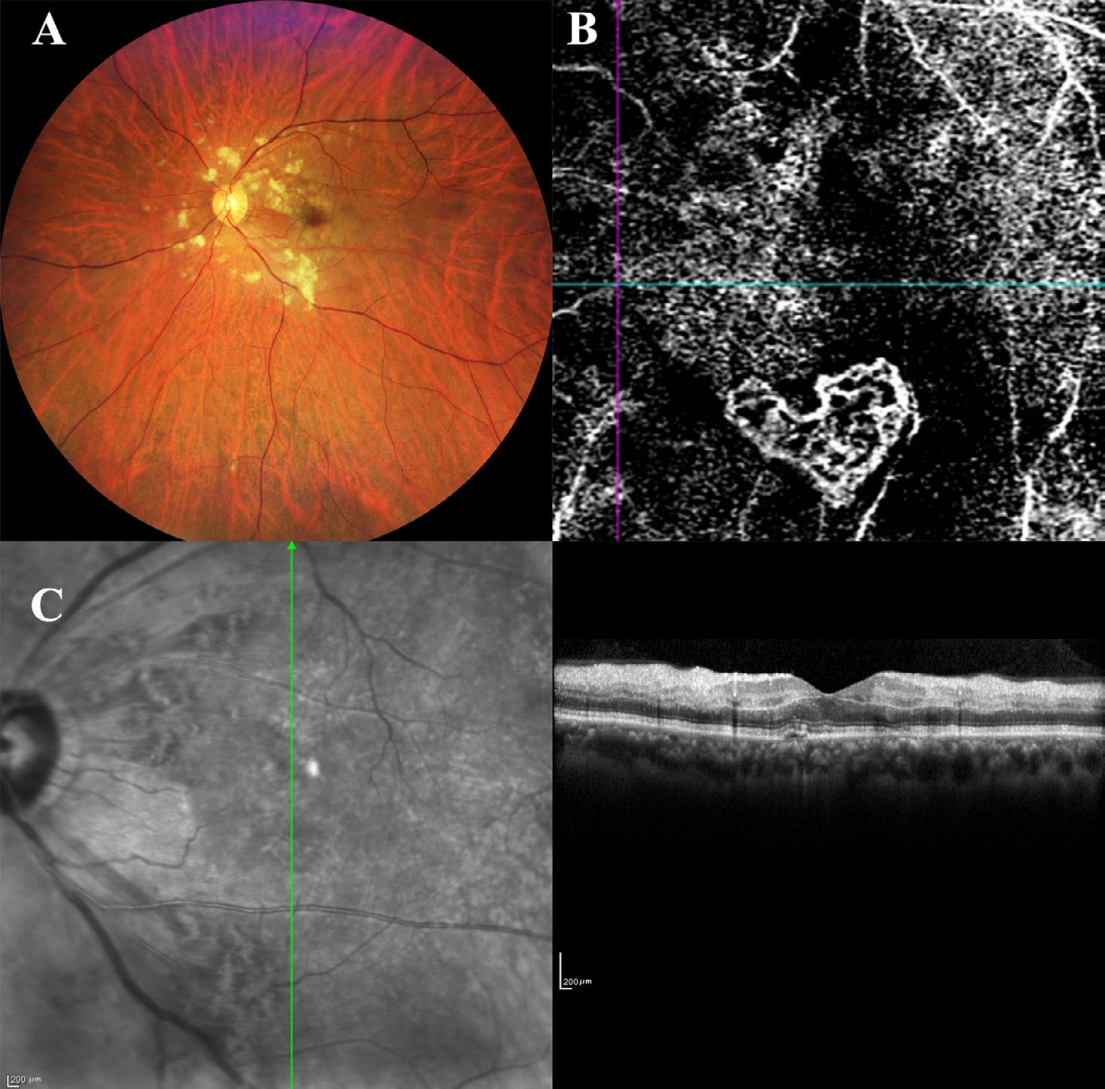
A 63‐year‐old man with heart‐shaped vascular intertwined nets of choroidal neovascularization. (A) The exudation accompanied by edema in the posterior pole. (B) The subretinal heart‐shaped vascular intertwined nets within the macular zone. (C) A hyper‐reflective lesion in the outer retina with a mild subretinal fluid in the OCT line scan passing through the fovea.

A 63‐year‐old male patient presented with a history of long‐standing hypertension, a prior cerebral infarction, and epilepsy. He reported a sudden, painless loss of vision in his left eye that had persisted for 3 days before seeking medical attention. During the initial ophthalmologic examination, the visual acuity in his left eye was found to be markedly reduced to 20/1000. Funduscopic examination revealed noticeable exudation and associated edema localized in the posterior pole of the retina (Figure 1A). OCTA was performed, which highlighted the presence of a distinct subretinal vascular network forming a heart‐shaped geometry intertwined within the macular area (Figure 1B). Furthermore, optical coherence tomography line scan, which was performed through the foveal region, showed a hyper‐reflective lesion situated in the outer retinal layers, accompanied by a mild accumulation of subretinal fluid (Figure 1C).

## 3 Differential Diagnosis, Investigations and Treatment

Based on these findings, a diagnosis of MNV secondary to hypertensive retinopathy was established. The patient's identified risk factors included advanced age, hypertension, and diabetes. In response to the diagnosis, the patient was treated with a series of intravitreal injections of Conbercept in the left eye. The injection is administered once every 3 months during the first 9 months, with the fourth injection depending on the progression of the condition.

## 4 Conclusion and Results (Outcome and Follow‐Up)

Over the course of a 1‐year follow‐up period, the patient demonstrated a significant improvement in visual acuity, which increased to 20/200. During this period, we did not observe any long‐term intraocular pressure elevation or subconjunctival hemorrhage, which are potential adverse events associated with Conbercept [4].

## 5 Discussion

Conbercept, an anti‐VEGF drug, received approval from NMPA in 2013 for the treatment of wAMD (Table 1), playing a significant role in improving vision. Two previous studies utilizing OCTA provide a theoretical framework for understanding and guiding the treatment strategies employed in our case. Tang et al. [28] conducted a study that distinguished between two distinct forms of inflammatory MNV: vascular loops and intertwined nets. Vascular loops are typically characterized by their small, irregular shapes and a circular flow geometry. These loops are composed predominantly of larger, prominent vessels without the presence of finer capillaries. In contrast, intertwined nets are distinguished by a more complex and elaborate vascular structure. This type of MNV is marked by a central core of feeder vessels surrounded by a network of smaller capillaries, often forming a dark ring or halo around the lesion. This description closely resembles the vascular geometry observed in our own study. Cheng et al. [27] further explored the effectiveness of Conbercept in treating the geometry of intertwined nets. Their findings indicated that Conbercept is capable of significantly reducing the area occupied by intertwined nets, which aligns well with the positive outcomes observed in our treatment regimen. This suggests that Conbercept is particularly effective for managing complex intertwined nets of MNV.

In addition to Conbercept, other approved anti‐VEGF drugs have also been employed in the treatment of different MNV vascular geometries. For example, Saoji et al. [29] found Bevacizumab has demonstrated notable therapeutic efficacy for certain neovascularization from parafoveal telangiectasia, but this is typically observed in cases where the MNV forms distinct clusters that exhibit discontinuity from the surrounding perifoveal vessels in OCTA imaging. This suggests that Bevacizumab may be most effective for specific MNV presentations that do not integrate seamlessly with the surrounding vascular structure. Furthermore, Marques et al. [30] revealed Aflibercept has shown remarkable effectiveness in managing glomerular‐like MNV, with substantial reductions in MNV area observed after a single intravitreal injection within a month. On the other hand, they also found Ranibizumab has proven to be especially effective in suppressing tree‐like MNV geometry.

From a pathophysiological perspective, different vascular geometries may represent distinct stages of vascular development or levels of lesion activity. Vascular loops may indicate an early sprouting stage of vessel formation [34, 35]. They are generally associated with immature vessels that are prone to leakage but may respond rapidly to anti‐VEGF therapy, as their incomplete vascular structure does not form a sufficient barrier against anti‐VEGF agents. Intertwined nets, in contrast, reflect a more complex and organized neovascular architecture with a dense capillary network. Moreover, the surrounding dark halo has been hypothesized to arise from blood sequestration during neovascular reactivation, and an enlarged dark halo is considered a sign of CNV growth [36, 37], although this interpretation remains controversial [37, 38, 39, 40, 41]. Tree‐like patterns could be thought to represent feeder vessel dominance with extensive branching and have been associated with chronicity and relative inactivity of the disease [42]. Finally, glomerular‐like morphologies may appear as hyperflow lesions in the outer retina, surrounded by a dark halo [43].

**FIGURE 2:**
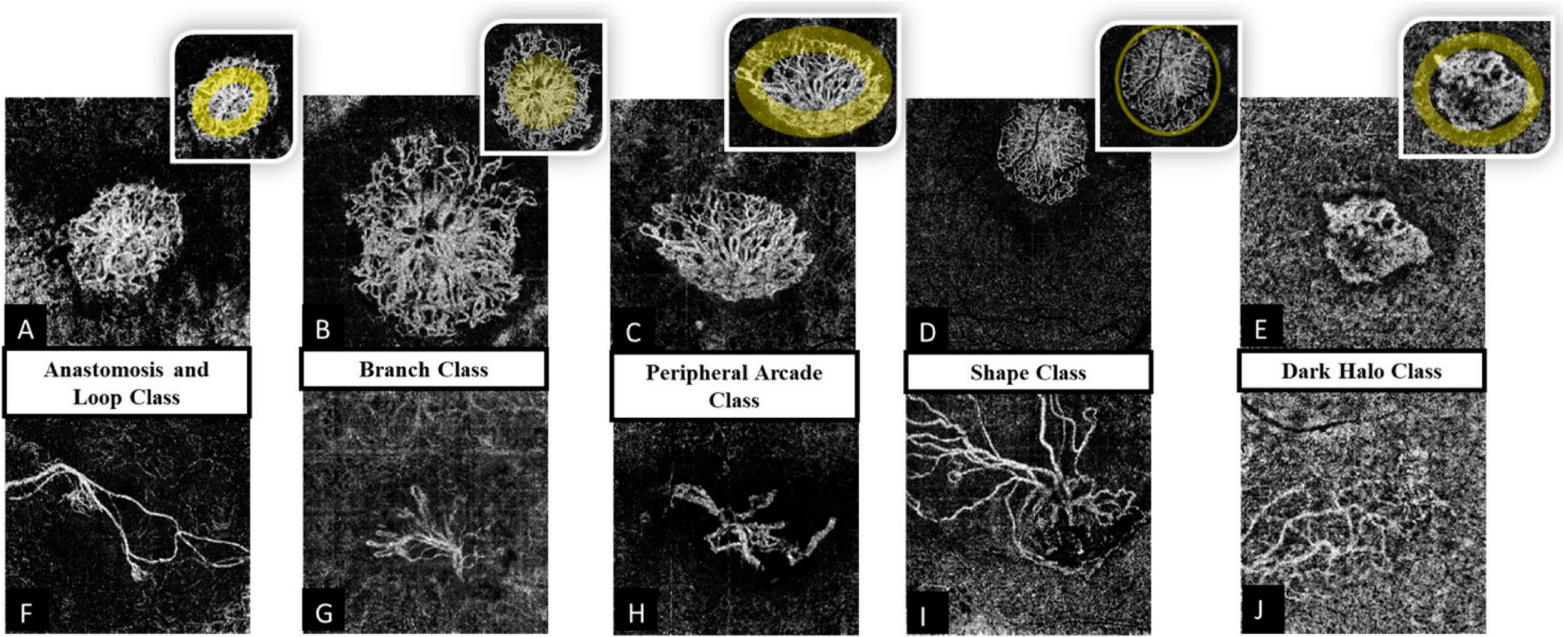
The presence and absence of anastomosis and loops (A and F), branching (B and G), peripheral arcade (C and H), shape (D and I), dark halo (E and J). The permission of reproducing is under the terms and conditions of the Creative Commons Attribution (CC BY) license from MDPI, Basel, Switzerland.

The classification of vascular geometry in MNV is fundamentally based on the imaging capabilities of OCTA. This approach is consistent with previous major classification systems and is highly valued due to OCTA's ability to visualize vascular geometry associated with MNV in both the outer retinal layer and the choriocapillaris. By enabling the identification of this geometry before and during treatment, OCTA contributes to a more profound understanding of the underlying pathophysiological mechanisms. This, in turn, facilitates the development of standardized protocols for image acquisition and interpretation, which are essential for both clinical practice and basic research. One of the early pioneers in classifying vascular geometry using OCTA was Cascos et al. [41], who proposed a set of five criteria based on OCTA images to assess MNV activity: anastomosis and loops, branching, peripheral arcade, shape, and dark halo. These criteria serve as a guideline for determining the activity level of MNV. Vali et al. [31] later provided clearer illustrations of typical and atypical OCTA images that correspond to these five criteria in a previous report (Figure 2, the permission of reproducing is under the terms and conditions of the Creative Commons Attribution (CC BY) license from MDPI, Basel, Switzerland and Ref. [31]). According to their findings, if the vessels in an OCTA image meet any three of these criteria, the MNV can be classified as active. While these criteria share similarities with the vascular geometry associated with certain anti‐VEGF drugs, as mentioned earlier, no study has yet fully established a comprehensive link between specific vascular geometry and particular anti‐VEGF therapies.

In our efforts to create a direct correlation between vascular geometry and the most suitable anti‐VEGF drugs using a simplified classification system, we aim to enhance the understanding of MNV for both doctors and patients. However, this approach encounters several challenges. The effectiveness of this classification system is heavily dependent on the precision of OCTA imaging in capturing the nuances of MNV and the clinician's ability to accurately interpret these spatial geometry. Historically, there have been discrepancies among clinicians in identifying and classifying the same MNV geometry, making standardization difficult. Recent advancements in artificial intelligence (AI) have the potential to overcome these challenges. AI‐driven algorithms for OCT and OCTA image recognition have significantly improved the accuracy of MNV classification. For instance, CNV‐Net [31], an AI‐based algorithm, offers automated segmentation of MNV areas and identification of MNV activity criteria within OCTA images. This tool can distinguish between different vascular geometry with a high degree of precision, applying the five classic criteria of MNV activity mentioned earlier. The integration of AI could thus enhance the reliability of this classification system and aid in the selection of the most appropriate anti‐VEGF drugs for specific MNV geometry.

Moreover, this case report also presents certain limitations, as it describes a single case and is consistent with most previous similar studies. As a result, it is difficult to use comparative experiments to reflect the differences among various anti‐VEGF agents in order to determine the optimal drug. Furthermore, it must be acknowledged that, at present, there remains a lack of in‐depth application of the distinct pharmacological mechanisms of anti‐VEGF therapies to explain treatment outcomes across different MNV geometries. This gap highlights the need for further molecular and biological investigations, along with a series of case–control studies and rigorous statistical analyses though some imaging parameters, such as central retinal thickness and exudate volume.

## 6 Conclusion

Although our case represents the first instance of connecting a specific vascular geometry to the broader efficacy of Conbercept, and suggests the potential benefits of Conbercept for targeting VEGF in certain MNV geometries over a 1‐year follow‐up period, further research is necessary. This research should focus on how to uniformly define different vascular geometries using AI and how to optimize treatment strategies accordingly. In conclusion, while this clinical case represents a single instance, it adds to the growing body of evidence suggesting that a deeper understanding of MNV pathology could lead to more effective use of anti‐VEGF drugs. The findings highlight the importance of personalized medicine in retinal disease management, offering potential pathways to enhance universal health coverage by tailoring treatments to individual patient needs. Refining treatment strategies based on specific vascular geometry not only improves patient outcomes but also contributes to global efforts to prevent vision‐related disabilities, reduce healthcare disparities, and promote overall well‐being.

## Author Contributions

**Shangkun Ou:** conceptualization, formal analysis, funding acquisition, investigation, methodology, project administration, resources, supervision, writing – review and editing. **Feiping Shi:** conceptualization, investigation, methodology, resources, writing – review and editing. **Minqing Cai:** formal analysis, writing – original draft, writing – review and editing. **Yiming Wu:** conceptualization, formal analysis, methodology, writing – original draft.

## Ethics Statement

The studies involving human participants were reviewed and approved by the Human Ethics Committee of Xiamen University Affiliated Xiamen Eye Center, in accordance with the Declaration of Helsinki.

## Consent

The patient/participant in Figure 1 provided written informed consent to participate in the study.

## Conflicts of Interest

The authors declare no conflicts of interest.

## Data Availability

The data that support the findings of this study are available from the corresponding author upon reasonable request.
